# A Treatise on the Animal Alkaloids, Cadaveric and Vital; or, the Ptomaines and Leucomaines Chemically, Physiologically, and Pathologically Considered in Relation to Scientific Medicine

**Published:** 1887-09

**Authors:** 


*A Treatise on the Animal Alkaloids, Cadaveric and Vital; or, the Ptomaines and Leucomaines Chemically, Physiologically, and Pathologically Considered in Relation to Scientific Medicine.* By A. M. BROWN, M.D. With an Introduction by PROFESSOR ARMAND GAUTIER. London: BAILLIÈRE, TINDALL, and COX. 1887.

*On the Animal Alkaloids, the Ptomaïnes, the Leucomaïnes, and Extractives, in their Pathological Relations; being a short Summary of Recent Researches as to the Origin of some Diseases by or through the Physiological Processes going on during Life.* By SIR WILLIAM AITKEN, Knt., M.D., F.R.S. London: H. K. LEWIS. 1887.

In the first book, Dr. Brown gives an able exposition of the researches on this subject from the earliest to the latest, with the intention of bringing before the English scientific student the many problems that these discoveries of animal alkaloids raise. The work opens with a lucid introduction by Professor Gautier, to whom the credit of being the first to discover the presence of these alkaloids in putrefactive albuminoid material is due. Shortly afterwards Selmi, the Italian medico-legal expert, found in the bodies of two persons, supposed to have been poisoned, an alkaloid hitherto unknown. It is not surprising that the announcement that organic toxic alkaloids, analogous to those of vegetable origin, were constantly formed during the processes of putrefaction was at first received with astonishment and even incredulity; but the subsequent researches of MM. Gautier, Pouchet, Hugounenq, and other observers successfully disposed of the objections raised.

The first part of the book gives an account of the methods of preparation, chemical properties, classification, and definitions of the ptomaïnes. As to their physiological actions, some are intensely toxic, one base being almost as powerful as the venom of cobra di capello; others are non-poisonous.

The last chapter of this part is occupied with a discussion of the measures to be adopted for determining whether an alkaloid extracted from the dead body—for instance, of a subject supposed to have been poisoned—is a ptomaïne, that is to say, has been elaborated during the process of putrefaction, or is a vegetable alkaloid introduced during life.

Obviously, to determine this with certainty is of paramount importance in medico-legal investigations.

The discovery of the ptomaïnes was followed by the detection of alkaloids in the urine, and then as a normal product of proteid metabolism; to these alkaloids, having an albuminoid origin in normal tissue transformation, Gautier gave the name of leucomaïnes: in the second part of the book these are treated of, so far as our present knowledge allows, in the same complete manner as the ptomaïnes. Since many of these leucomaïnes are highly poisonous—it has been calculated that the alkaloids contained in the twenty-four hours' fæces of a healthy man are sufficient to kill him—we accordingly find that there is abundant provision for their destruction and excretion. They are destroyed by oxygenation in the blood, and excreted through the emunctories. The recent experiments on peptones show that the liver especially has for one of its functions the destruction of harmful products of albuminoid metamorphosis, and the prevention of their entrance into the general circulation.

We can see how fruitful in the production of disease the ingestion of these bodies may become, and that the danger may be from within, an auto-infection, or from without, through unhealthy surroundings or bad food, &c. M. Peter considers that there is a group of symptoms produced by auto-infection from accumulation of the extractives in the system, especially produced by over-fatigue, and this "fever of over-taxation" forms the lowest member of a series which typhoid and typhus fevers complete. In other words, this "auto-typhusation" may occur in a more virulent form than as a mere "fatigue-fever," and may then be communicable to others. Space will not allow us however to enter into a discussion of the many interesting questions raised in the third part of the book, which deals with the relation of the ptomaïnes and leucomaïnes to scientific medicine.

Both Dr. Brown and Sir William Aitken vigorously attack those who uphold the view that the virus of many of the specific diseases is a micro-organism. Although diseases such as rheumatism, gout, and perhaps cholera are most satisfactorily explained by an auto- or hetero-infection from alkaloids, the relation of a micro-organism as the cause of others—it may be through products of the metabolism of these microbes—is too firmly established to be overthrown without further evidence. We do not think that the average medical man will echo Professor Aitken's joy at the prospect of being delivered from the hands of the bacteriologist into those of the expert in the chemistry of the alkaloids; the former's yoke has been grievous, but at least rods of various colours are more entertaining than pages of bewildering chemical formulæ.

Dr. Brown's volume concludes with a bibliography brought up to date. We do not like to find fault with so excellent a book; but if the author had given more care to the composition of his sentences, much slipshod writing would have been corrected.

Sir William Aitken's book can be cordially recommended to all who wish to have an accurate account in small compass of the recent discoveries of animal alkaloids. It consists of two lectures delivered to his class at Netley, with the object both of acquainting them with the existing knowledge of the subject, and of stimulating them by their own exertions to further extend it. In a clear and concise manner, Professor Aitken admirably brings together and compares the work of the chief observers, while at the same time, far from being a mere abstract, his writing is full of original ideas and pregnant suggestions, and of well-reasoned argument founded on facts and theories both new and old.

